# Supplementary material for: Inflammatory Endotype-Associated Airway Resistome in Chronic Obstructive Pulmonary Disease
Source: Microbiol Spectr. 2022 Mar 21;10(2):e02593-21. doi: 10.1128/spectrum.02593-21 (PMC9045194; doi:10.1128/spectrum.02593-21)
Supplement: SUPPLEMENTAL FILE 1 — Supplemental material. Download SPECTRUM02593-21_Supp_1_seq6.pdf, PDF file, 1.1 MB [file spectrum02593-21_supp_1_seq6.pdf]

Inflammatory Endotype Associated Airway Resistome in Chronic Obstructive Pulmonary Disease

Supplementary document

Supplementary Methods

Patient recruitment

Sputum samples of 99 stable COPD patients were collected in the First Affiliated Hospital of Guangzhou Medical University and Shenzhen People’s Hospital. The study was approved and supervised by the ethics committee of the two centers (reference number: 2017-22 and KY-LL-2020294-01), following the Helsinki declaration and good clinical practices. All COPD patients met the diagnostic criteria according to GOLD guideline and were also assessed for symptoms and exacerbation frequency. GOLD classification for disease severity (stage I-IV) was assigned to COPD patients based on pulmonary function test. For COPD patients, the inclusion criteria were: (1) age > 40 years; and (2) confirmed diagnosis of COPD according to the GOLD guideline (post-bronchodilator forced expiratory volume in 1s [FEV1]/forced vital capacity [FVC] ratio < 0.7). The exclusion criteria were: (1) physician-diagnosis of asthma or significant respiratory disease other than COPD; (2) COPD exacerbation within 4 weeks of enrollment; (3) history of lung surgery and tuberculosis; (4) diagnosis of cancer; (5) blood transfusion within 4 weeks of enrollment; (6) diagnosis of autoimmune diseases; (7) enrollment in a blinded drug trial; and (8) short-term antibiotic usage within 4 weeks of enrollment. Informed consent was obtained from all participants. Five participants without long-term antibiotic use information recorded were excluded from analyses. All remaining 94 participants were free of long-term antibiotic use.

Quality control of sputum samples

Induced sputum were obtained for all subjects and quality-controlled upon collection. Briefly, sputum plugs which contained the most viscous material were picked up in a petri dish and isolated from saliva. The selected sputum plugs were prepared for cytology by dilution with 0.1% dithiothreitol (DTT) solution and filtered through 48 µm nylon-mesh filter according to standardized sputum processing protocol(1). The numbers of total cells, squamous epithelial cells and leukocytes were counted and recorded. Sputum specimens with squamous epithelial cells:leukocytes<1:2.5 were considered unlikely to be contaminated with oropharyngeal flora and acceptable for downstream experiments(2).

Metagenomic sequencing

Bacterial genomic DNA was extracted from selected, quality-controlled sputum plugs using Qiagen DNA Mini kit and subject to ultra-deep sequencing using Illumina NovaSeq (targeted >30G sequences per sample). The raw sequencing reads for metagenome were processed using Sunbeam pipeline(3), in which quality filtering was performed using Cutadapt (v2.5)(4), sequences with low complexity were filtered using Komplexity(3), and host reads were filtered by mapping to human genome hg38 using BWA (v0.7.17)(5). The remaining non-human reads were subject to the resistome analyses as detailed below. Four reagent negative controls were included for sequencing. These negative controls were DNA extraction blanks in which nuclease-free water was used for genomic DNA extraction, library preparation and sequencing, performed side-by-side with the sputum samples. Bacterial species identified in at least two of four reagent controls with relative abundance greater than 0.001 were considered potential contaminants and excluded from downstream analyses (**Table S6**).

Quantification of antibiotic resistant genes (ARGs) in metagenomic data

The non-human reads were used to search for ARGs following ARGs-OAP v2.0 pipeline as described by Yin et al.(6), where parameters for similarity-based identification include alignment length cut-off of 75 nucleotides, alignment e value cut-off of 10^{-7}, and alignment identity of 80%. The perl script was customized to calculate ARG abundance as rpkm:

$$\text{rpkm} = \frac{\#\ of\ ARG{-}like\ shot\ reads}{\frac{reference\ ARG\ length}{1000} \times \frac{\#\ of\ reads\ in\ metagenome}{1000000}}$$

Taxonomic annotation for ARG-like sequencing reads

The ARG-like sequencing reads were extracted from metagenomic data and the taxonomic information for each ARG-like read was obtained using Kraken 2 with default settings and the Minikraken2_v2_8GB database released in April 2019(7). The contribution of each taxon to a specific ARG was quantified using a custom R script by calculating the read count of the ARG from the corresponding taxon divided by the total read count of that ARG.

Host transcriptomic sequencing

The remaining sputum was subject to cell and supernatant isolation according to previous protocol(1, 8). RNA was extracted from sputum cell using Qiagen RNease Mini kit for mRNA-Seq using Illumina NovaSeq. The raw reads for host transcriptome were quality filtered using Cutadapt (v2.5)(4) and aligned to human genome hg38 using Hisat2 (v2.1.0)(9). The gene count matrix was obtained using RSEM (v1.3.1)(10), normalized using DESeq2 (v1.20)(11) and subject to co-abundance clustering using WGCNA (v1.69)(12). Significantly enriched pathways of each module were identified using MetaBase (Clarivate Analytics, FDR<0.05).

Sputum Inflammatory Mediators

A panel of 47 sputum mediators (BLC, Eotaxin, Eotaxin-2, CXCL11, CXCL10, CCL2, CCL3, CCL4, CCL5, CCL13, CCL17, G-CSF, GM-CSF, I-309, ICAM-1, IFNg, IL-1a, IL-1ra, IL-1b, IL-2, IL-4, IL-5, IL-6, IL-6R, IL-7, IL-8, IL-10, IL-12p40, IL-12p70, IL-13, IL-15, IL-16, IL-17, IL-21, MCSF, MIG, MIP-1d, MMP-8, MMP-9, PDGF-AB, Procalcitonin, TNFa, TNFb, TNFRI, TNFRII, TIMP-1, TIMP-2) were measured in a subset of 43 patients with extra sputum samples available using custom antibody microarray (Human Cytokine Antibody Microarray slides; RayBiotech Inc., Norcross, GA, United States)(13).

Sputum culture and antimicrobial susceptibility testing

After liquefaction of sputum samples, 100ul liquid were cultured in Columbia blood agar plate and chocolate agar plate (Autobio, China). Four-zone method were performed for isolation of microbial colonies. Agar plates were incubated for 48 h under a condition of 37° and 5% CO_2 in the Reach-IN CO_2 Incubator (Thermo scientific, USA). The taxonomy of all colonies that appeared to be morphologically distinct were identified by Vitek MALDI-TOF mass spectrometry (bioMérieux, German).

For each isolated bacterium, antimicrobial susceptibility testing were performed by Vitek-2 Compact Automatic System (bioMérieux, German), to generate the Minimum Inhibitory Concentration (MIC) level to a list of 33 antibiotic drugs. The sensitivity of bacterial isolates to Doxycycline and Minocycline was tested using broth microdilution method. The MIC of each bacterium to each drug was determined as sensitive (S), intermediate (I) and resistance (R) according to the National Clinical Laboratory Standardization Committee (CLSI) of the MIC (M100, performance standards for antimicrobial

susceptibility testing, 30th edition). Quality control strains included *Staphylococcus aureus* ATCC 29213 and *Escherichia coli* ATCC 25922.

Statistical analysis

Procrustes analysis was performed to assess the correlation between the resistome and microbiota taxonomic composition, based on the coordinate matrices of both datasets generated by principal coordinate analysis using Bray-Curtis dissimilarity indices, using the *procrustes* function in R vegan package. Unsupervised clustering was performed on the ARG profiles by the Ward's method using hclust function in R stat package. The association of patient demographic and clinical features with the resistome was assessed by PERMANOVA using adonis function in R vegan package. The association between each ARG and neutrophil and eosinophil percentages was assessed by Spearman correlation in R. An all-against all correlation analysis was performed between all ARGs and host transcriptomic modules and sputum inflammatory mediators using HAllA (Hierarchical All-against-All association testing)(14). The association between host transcriptomic modules and sputum inflammatory mediators with the resistome profile in each inflammatory endotype was assessed by canonical correspondence analysis in R vegan package. The Benjamin-Hochberg method was used to calculate false discovery rate (FDR) adjusted P-value whenever applicable(15).

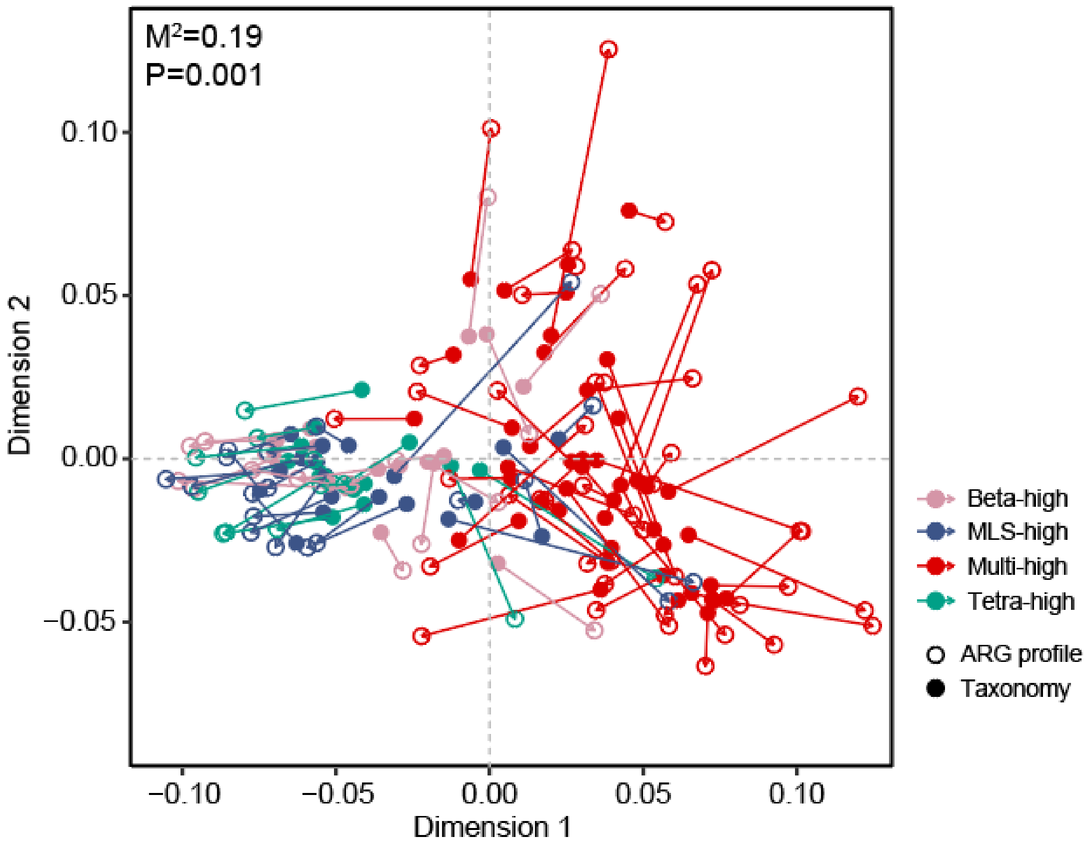

Figure S1. Procrustes analysis based on Bray-Curtis dissimilarity matrices showed a significant correlation between the airway resistome and microbiome taxonomic composition.

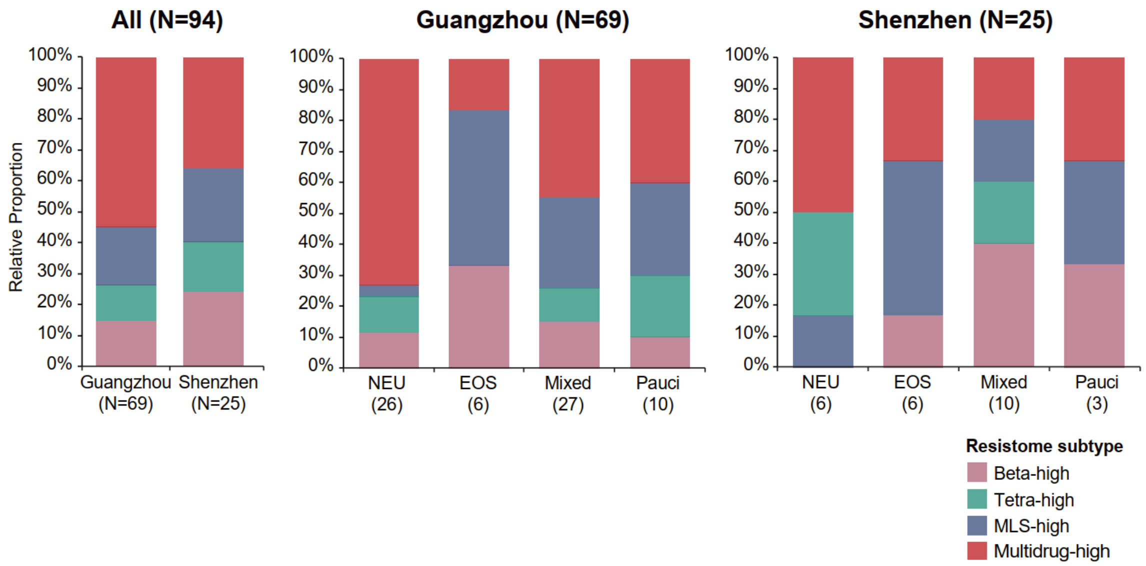

Figure S2. The distribution of the resistome subtypes in COPD participants with different inflammatory endotypes, in Guangzhou and Shenzhen cohort separately.

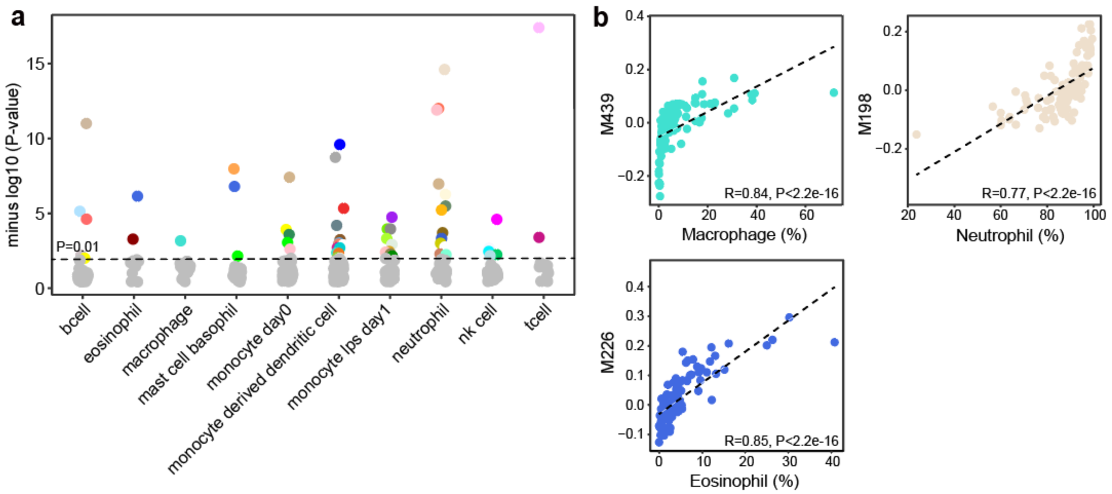

Figure S3. Enrichment analysis for immune cell marker genes using sputum transcriptomic data. a) The enrichment of transcriptomic co-abundance modules to marker genes for specific cell types, according to Peters et al.(16). The minus log10 P-value was shown for the enrichment of each module in each cell type. For modules with P<0.01, they were colored according to the color codes of the modules in WGCNA. **b)** For modules most enriched for the marker genes for macrophage, neutrophil and eosinophil, their expression levels were highly correlated with sputum differential cell count of the corresponding cell type.

Table S1. Major demographic and clinical features of all 94 COPD participants. The participants are grouped by clinical sites (Guangzhou and Shenzhen) and four different resistome subtypes (Beta-lactam-high, MLS-high, Multidrug-high, Tetracycline-high).

Feature	Site		P-value#	Resistome subtype				P-value##
	Guangzhou (N=69)	Shenzhen (N=25)		Beta-lactam-high (N=16)	MLS-high (N=19)	Multidrug-high (N=47)	Tetracycline-high (N=12)	
Age	66.1±9.1	66.5±6.4	0.888	63.2±10.5	63.9±7.8	67.8±8.4	67.8±4.3	0.14
Gender (F/M)	4/65	2/23	0.149	1/15	1/18	3/44	1/11	0.989
Current Smoking (Y/N)	22/47	10/15	0.463	6/10	8/11	13/34	5/7	0.615
GOLD (A/B/C/D)	31/26/2/10	16/2/3/4	0.022	9/3/1/3	9/7/1/2	22/15/3/7	7/3/1/1	0.984
Freq Exacerbator (Y/N)*	12/57	7/18	0.257	4/12	3/16	10/37	2/10	0.899
ICS usage (Y/N)	37/32	8/17	0.064	8/8	12/7	30/17	4/8	0.235
LABA usage (Y/N)	37/32	8/17	0.064	7/9	12/7	31/16	4/8	0.126
LAMA usage (Y/N)	35/34	9/16	0.206	6/10	10/9	27/20	8/4	0.429
pre-FEV1	1.6±0.8	1.3±0.6	0.207	1.7±0.8	1.3±0.6	1.6±0.9	1.4±0.6	0.428
pre-FEV1 perc	60.1±28.7	49.7±20.5	0.213	60.7±28.8	44.6±17.3	61.3±29.6	57.4±23.2	0.183
pre-FEV1/FVC	0.5±0.2	0.5±0.1	0.687	0.5±0.2	0.4±0.1	0.5±0.2	0.5±0.1	0.085
pre-FVC	3.1±0.9	2.7±0.8	0.057	3.0±0.9	2.9±0.9	3.0±0.9	2.8±0.9	0.776
post-FEV1	1.6±0.8	1.4±0.6	0.311	1.8±0.8	1.4±0.6	1.6±0.8	1.6±0.6	0.417
post-FEV1 perc	61.5±27.2	49.2±17.6	0.263	62.2±29.0	52.1±18.9	61.7±28.2	61.0±17.4	0.278
post-FEV1/FVC	0.5±0.1	0.4±0.1	0.517	0.5±0.2	0.5±0.1	0.5±0.1	0.5±0.1	0.132
post-FVC	3.2±0.9	3.1±0.8	0.217	3.2±1.0	2.9±0.9	3.1±0.9	3.0±0.8	0.823
CAT score	8.7±4.7	5.0±5.6	<0.001	6.3±3.5	9.3±6.2	7.6±4.9	7.8±5.8	0.594
mMRC	1.4±1.0	1.2±1.1	0.261	1.6±1.2	1.3±0.9	1.3±1.0	1.5±0.9	0.753
Neutrophil (%)	85.9±12.6	79±22.2	0.112	80.8±19.4	84.2±10.4	84.9±17.8	85.2±7.1	0.412
Macrophage (%)	8.5±12.0	14.1±22.5	0.168	11.8±18.3	8.8±10.5	9.4±17.6	11.2±7.4	0.171
Eosinophil (%)	5.0±7.0	5.5±5.9	0.326	6.4±9.5	6.0±6.1	5.0±6.6	2.9±2.5	0.209
Lymphocyte (%)	0.7±0.5	1.3±1.4	0.013	0.9±0.6	1.0±1.3	0.7±0.8	0.8±0.7	0.467

Continuous data are presented as mean±SD unless otherwise stated. #P-value was calculated using Fisher exact test for categorical variables and using Wilcoxon rank-sum test for continuous variables. ## P-value was calculated using Fisher exact test for categorical variables and using Kruskal-Wallis test for continuous variables. * Frequent exacerbator was defined as exacerbation event > = 2/last year.

Table S2. Association of demographic and clinical features with the airway resistome in all 94 COPD participants. The association was assessed by PERMANOVA. The R-squared and FDR P-value were shown for each feature.

**Feature**	**R-squared**	**FDR P-value**
Inflammatory endotype	0.087	0.043
Neutrophil (%)	0.039	0.082
post-FVC	0.037	0.061
post-FEV1	0.032	0.087
Eosinophil (%)	0.032	0.112
LABA usage	0.032	0.121
ICS usage	0.030	0.113
BMI	0.029	0.184
GOLD (A/B/C/D)	0.029	0.157
Clinical site	0.026	0.320
post-FEV1% pred	0.026	0.291
mMRC	0.026	0.260
Macrophage	0.025	0.318
Antibiotic use prior 6m	0.024	0.331
CAT score	0.023	0.358
Age	0.021	0.450
Smoking	0.019	0.604
post-FEV1/FVC	0.019	0.699
LAMA usage	0.018	0.668
Lymphocyte (%)	0.018	0.694
Exacerbation frequency	0.017	0.750
Gender	0.014	0.882

Table S3. The results for sputum culture and antimicrobial susceptibility testing for the subset of 33 participants with extra sputum samples available. Only the results for drug resistance (R) are shown, with the cultured bacterial species, tested antibiotic drug and MIC indicated. The resistome type was summarized for each subject based on the drug resistant pattern of bacterial species cultured from the subject.

Subject ID	Inflammatory endotype	Resistome type	Tetracycline	Beta-lactam	Macrolide	Others
J059	NEU	Macrolide			Streptococcus peroris:Erythromycin(2R)	
J007	NEU	Multidrug	Acinetobacter baumannii:Doxycycline(>=16R), Minocycline(>=16R)	Acinetobacter baumannii:Aztreonam(>=64R)	Streptococcus oralis:Erythromycin(2R)	Acinetobacter baumannii:Piperacillin(16R),Ceftazidime(>=64R),Ciprofloxacin(>=4R),Levofloxacin(>=8R)
J027	NEU	Multidrug		Streptococcus salivarius:Benzylpenicillin(2R)	Streptococcus salivarius:Erythromycin(2R); Streptococcus parasanguinis:Erythromycin(2R)	Streptococcus salivarius:Levofloxacin(>=8R)
K079	NEU	Multidrug	Pseudomonas aeruginosa:Tigecycline(>=8R)	Pseudomonas aeruginosa:Ampicillin(8R)	Streptococcus parasanguinis:Erythromycin(1R)	Streptococcus parasanguinis:Levofloxacin(>=8R)
K180	NEU	Multidrug		Klebsiella pneumonia:Cefuroxime(4R)	Streptococcus salivarius:Erythromycin(>=8R)	Streptococcus salivarius:Clindamycin(>=4R)
K069	NEU	Multidrug			Streptococcus oralis:Erythromycin(2R)	Streptococcus oralis:Levofloxacin(>=8R)

Y001	NEU	Multidrug			Streptococcus parasanguinis:Erythromycin(>=8R)	Streptococcus parasanguinis:Levofloxacin(>=8R)
J011	NEU	Multidrug			Rothia dentocariosa:Erythromycin(>=8R)	Rothia dentocariosa:Levofloxacin(>=8R)
K097	NEU	Multidrug	Pseudomonas aeruginosa:Doxycycline(>=16R)	Pseudomonas aeruginosa:Ampicillin(>=8R)		Pseudomonas aeruginosa:Levofloxacin(>=8R)
J093	NEU	None				
K072	NEU	None				
J043	NEU	Others				Corynebacterium argentoratense:Levofloxacin(8R)
K117	NEU	Others				Streptococcus salivarius:Clindamycin(>=4R)
J005	EOS	Macrolide			Streptococcus gordonii:Erythromycin(4R)	
J095	EOS	Macrolide			Rothia dentocariosa:Erythromycin(>=8R)	
J035	EOS	Macrolide			Granulicatella adiacens:Erythromycin(>=8R)	
K174	EOS	None				
K085	EOS	None				

K056	Mixed	Multidrug	Pseudomonas aeruginosa:Ampicillin(>=8R)		Rothia mucilaginosa:Levofloxacin(>=8R),Linezolid(>=8R)
K111	Mixed	Multidrug	Pseudomonas aeruginosa:Ampicillin(>=8R)		Pseudomonas aeruginosa:Levofloxacin(>=8R)
1040	Mixed	None			
J003	Mixed	None			
J060	Mixed	None			
K084	Mixed	None			
K082	Mixed	Others			Streptococcus oralis:Levofloxacin(>=8R)
K003	Pauci	Macrolide		Streptococcus mitis:Erythromycin(2R)	
K099	Pauci	Macrolide		Streptococcus oralis:Erythromycin(1R)	
K095	Pauci	Macrolide		Rothia mucilaginosa:Erythromycin(1R)	
K064	Pauci	Macrolide		Rothia dentocariosa:Erythromycin(2R)	
K172	Pauci	Multidrug		Rothia dentocariosa:Erythromycin(>=8R)	Rothia dentocariosa:Levofloxacin(>=8R)
1055	Pauci	None			
K067	Pauci	None			
K081	Pauci	None			

Table S4. Correlation of ARGs with neutrophil and eosinophil percentages. For each ARG, its antibiotic class, Spearman's rho and FDR P-value in correlation with neutrophil and eosinophil percentages are shown.

ARGs	Antibiotic class	NEU Cor	NEU FDR	EOS Cor	EOS FDR	Cor cell type
TEM-29	beta-lactam	0.448	0.000	-0.099	0.281	NEU
OXA-348	beta-lactam	0.417	0.000	-0.041	0.648	NEU
ant(2'')-I	aminoglycoside	0.401	0.000	-0.093	0.309	NEU
tetR	tetracycline	0.369	0.000	-0.089	0.329	NEU
sul1	sulfonamide	0.350	0.000	-0.106	0.250	NEU
mdtH	multidrug	0.326	0.000	0.073	0.425	NEU
aadA	aminoglycoside	0.323	0.000	-0.086	0.346	NEU
mexI	multidrug	0.292	0.001	-0.011	0.899	NEU
multidrug_ABC_transporter	multidrug	0.268	0.002	-0.027	0.758	NEU
aph(3''')-III	aminoglycoside	0.266	0.003	-0.050	0.579	NEU
mdtB	multidrug	0.261	0.003	0.011	0.904	NEU
mdtC	multidrug	0.254	0.004	0.004	0.964	NEU
rosA	fosmidomycin	0.247	0.005	0.073	0.423	NEU
mdtD	multidrug	0.240	0.006	-0.007	0.934	NEU
tetQ	tetracycline	0.227	0.010	-0.152	0.103	NEU
ermF	macrolide-lincosamide-streptogramin	0.208	0.018	-0.102	0.267	NEU
emrB	multidrug	0.204	0.020	-0.027	0.760	NEU
cmeB	multidrug	0.200	0.023	-0.105	0.253	NEU
OXA-60	beta-lactam	0.190	0.031	-0.019	0.826	NEU
OXA-22	beta-lactam	0.186	0.035	-0.043	0.629	NEU
oprC	multidrug	0.172	0.050	-0.066	0.464	NEU
adeK	multidrug	-0.179	0.042	-0.120	0.191	NEU
mdtL	multidrug	0.130	0.134	0.187	0.044	EOS
mdtM	multidrug	0.082	0.341	0.219	0.018	EOS
transcriptional regulatory protein CpxR cpxR	unclassified	0.052	0.533	0.194	0.036	EOS
bacterial regulatory protein LuxR	unclassified	0.049	0.559	0.187	0.043	EOS
macB	macrolide-lincosamide-streptogramin	0.049	0.559	0.325	0.000	EOS
ompF	multidrug	0.048	0.566	0.180	0.050	EOS
OXA-358	beta-lactam	0.043	0.606	0.203	0.029	EOS
bicyclomycin-multidrug_efflux_protein_bcr	multidrug	0.042	0.617	0.253	0.006	EOS

chloramphenicol exporter	chloramphenicol	0.037	0.661	0.188	0.042	EOS
DNA-binding_protein_H-NS	unclassified	0.018	0.825	0.232	0.012	EOS
omp36	multidrug	0.013	0.873	0.211	0.023	EOS
cAMP-regulatory protein	unclassified	0.011	0.894	0.192	0.038	EOS
lnuA	macrolide-lincosamide-streptogramin	0.011	0.896	0.295	0.001	EOS
TolC	multidrug	0.008	0.917	0.207	0.026	EOS
vanS	vancomycin	-0.020	0.811	0.277	0.003	EOS
PBP-1B	beta-lactam	-0.044	0.600	-0.208	0.025	EOS
metallo-beta-lactamase	beta-lactam	-0.059	0.487	-0.187	0.044	EOS
rifampin monooxygenase	rifamycin	-0.068	0.424	0.281	0.002	EOS
TEM-91	beta-lactam	-0.084	0.326	-0.187	0.044	EOS
PBP-1A	beta-lactam	-0.088	0.307	-0.186	0.045	EOS

Table S5. Correlation of ARGs with host transcriptome modules and inflammatory mediators in neutrophil-predominant and eosinophil-predominant COPD participants. For each ARG, its antibiotic class, correlated host omic type (transcriptome module or inflammatory mediator) and host omic feature, Spearman's rho and FDR P-value are shown.

Endotype	ARGs	Antibiotic class	Host omic feature	Host omic type	Correlation	FDR P-value
NEU	aadA	aminoglycoside	IL-15	Mediator	0.552	0.000
NEU	aadA	aminoglycoside	IL-7	Mediator	0.528	0.000
NEU	aadA	aminoglycoside	TNFa	Mediator	0.436	0.003
NEU	cmeB	multidrug	BLC	Mediator	0.506	0.001
NEU	cmeB	multidrug	M334: HIF1 activation	Transcriptome	0.292	0.007
NEU	cmeB	multidrug	M338: T cell receptor signaling pathway	Transcriptome	0.282	0.009
NEU	cmeB	multidrug	M379: ROS and arachidonic acid metabolites production	Transcriptome	0.349	0.001
NEU	cmeB	multidrug	M468: BAFF-induced non-canonical NF-kB signaling	Transcriptome	0.321	0.003
NEU	emrB	multidrug	MMP-8	Mediator	0.399	0.008
NEU	emrB	multidrug	MMP-9	Mediator	0.404	0.007
NEU	emrB	multidrug	M276: Bradykinin / Kallidin maturation	Transcriptome	0.437	0.000
NEU	emrB	multidrug	M280: Apoptosis and proliferation of epithelial cells	Transcriptome	0.317	0.003
NEU	emrB	multidrug	M281: Ubiquitin pathway	Transcriptome	-0.286	0.008
NEU	ermF	macrolide-lincosamide-streptogramin	BLC	Mediator	0.559	0.000

NEU	ermF	macrolide-lincosamide-streptogramin	IL-16	Mediator	0.573	0.000
NEU	ermF	macrolide-lincosamide-streptogramin	IP-10	Mediator	0.482	0.001
NEU	ermF	macrolide-lincosamide-streptogramin	M234: Androgen Receptor nuclear signaling	Transcriptome	-0.228	0.036
NEU	mdtB	multidrug	M173: Chromosome separation	Transcriptome	-0.300	0.005
NEU	mdtB	multidrug	M175: Oxidative stress and apoptosis in airway epithelial cells	Transcriptome	-0.328	0.002
NEU	mdtB	multidrug	M276: Bradykinin / Kallidin maturation	Transcriptome	0.291	0.007
NEU	mdtB	multidrug	M280: Apoptosis and proliferation of epithelial cells	Transcriptome	0.309	0.004
NEU	mdtB	multidrug	M281: Ubiquitin pathway	Transcriptome	-0.295	0.006
NEU	mdtB	multidrug	M482: Cell adhesion and migration	Transcriptome	0.430	0.000
NEU	mdtB	multidrug	M497: Histone deacetylases	Transcriptome	0.354	0.001
NEU	mdtC	multidrug	M281: Ubiquitin pathway	Transcriptome	-0.317	0.003
NEU	mdtC	multidrug	M482: Cell adhesion and migration	Transcriptome	0.406	0.000
NEU	mdtC	multidrug	M497: Histone deacetylases	Transcriptome	0.297	0.006
NEU	mdtD	multidrug	IL-15	Mediator	0.321	0.036
NEU	mdtD	multidrug	MMP-8	Mediator	0.481	0.015
NEU	mdtD	multidrug	M173: Chromosome separation	Transcriptome	-0.280	0.010
NEU	mdtD	multidrug	M281: Ubiquitin pathway	Transcriptome	-0.284	0.008
NEU	mdtH	multidrug	M173: Chromosome separation	Transcriptome	-0.316	0.003

NEU	mdtH	multidrug	M175: Oxidative stress and apoptosis in airway epithelial cells	Transcriptome	-0.284	0.009
NEU	mdtH	multidrug	M281: Ubiquitin pathway	Transcriptome	-0.400	0.000
NEU	mdtH	multidrug	M482: Cell adhesion and migration	Transcriptome	0.482	0.000
NEU	mdtH	multidrug	M497: Histone deacetylases	Transcriptome	0.330	0.002
NEU	multidrug_ABC_transporter	multidrug	M173: Chromosome separation	Transcriptome	-0.282	0.009
NEU	multidrug_ABC_transporter	multidrug	M175: Oxidative stress and apoptosis in airway epithelial cells	Transcriptome	-0.292	0.007
NEU	multidrug_ABC_transporter	multidrug	M280: Apoptosis and proliferation of epithelial cells	Transcriptome	0.297	0.006
NEU	OXA-22	beta-lactam	M497: Histone deacetylases	Transcriptome	0.307	0.004
NEU	OXA-60	beta-lactam	IL-7	Mediator	0.349	0.022
NEU	OXA-60	beta-lactam	M173: Chromosome separation	Transcriptome	-0.284	0.009
NEU	OXA-60	beta-lactam	M175: Oxidative stress and apoptosis in airway epithelial cells	Transcriptome	-0.313	0.004
NEU	OXA-60	beta-lactam	M276: Bradykinin / Kallidin maturation	Transcriptome	0.292	0.007
NEU	OXA-60	beta-lactam	M280: Apoptosis and proliferation of epithelial cells	Transcriptome	0.397	0.000
NEU	OXA-60	beta-lactam	M281: Ubiquitin pathway	Transcriptome	-0.306	0.004
NEU	OXA-60	beta-lactam	M295: Th17 cytokines in COPD	Transcriptome	0.296	0.006
NEU	OXA-60	beta-lactam	M482: Cell adhesion and migration	Transcriptome	0.342	0.001
NEU	rosA	fosmidomycin	IL-2	Mediator	0.420	0.005
NEU	rosA	fosmidomycin	IL-7	Mediator	0.391	0.009
NEU	rosA	fosmidomycin	M175: Oxidative stress and apoptosis in airway epithelial cells	Transcriptome	-0.298	0.006
NEU	rosA	fosmidomycin	M281: Ubiquitin pathway	Transcriptome	-0.330	0.002
NEU	rosA	fosmidomycin	M482: Cell adhesion and migration	Transcriptome	0.396	0.000

NEU	tetQ	tetracycline	BLC	Mediator	0.473	0.001
NEU	tetQ	tetracycline	IL-16	Mediator	0.613	0.000
NEU	tetQ	tetracycline	MMP-8	Mediator	0.492	0.001
NEU	tetQ	tetracycline	MMP-9	Mediator	0.399	0.008
NEU	tetQ	tetracycline	TIMP-1	Mediator	0.399	0.008
NEU	tetQ	tetracycline	M234: Androgen Receptor nuclear signaling	Transcriptome	-0.304	0.005
NEU	tetQ	tetracycline	M334: HIF1 activation	Transcriptome	0.285	0.008
NEU	tetQ	tetracycline	M338: T cell receptor signaling pathway	Transcriptome	0.284	0.008
NEU	tetQ	tetracycline	M379: ROS and arachidonic acid metabolites production	Transcriptome	0.418	0.000
NEU	tetQ	tetracycline	M468: BAFF-induced non-canonical NF-kB signaling	Transcriptome	0.279	0.010
EOS	bacterial regulatory protein LuxR	unclassified	IL-13	Mediator	0.447	0.003
EOS	bacterial regulatory protein LuxR	unclassified	IL-2	Mediator	0.461	0.002
EOS	bacterial regulatory protein LuxR	unclassified	IL-5	Mediator	0.393	0.009
EOS	bacterial regulatory protein LuxR	unclassified	M23: GTP-XTP metabolism	Transcriptome	-0.298	0.006
EOS	bacterial regulatory protein LuxR	unclassified	M480: Role of inhibition of WNT signaling in the progression of lung cancer	Transcriptome	0.282	0.009
EOS	bicyclomycin-multidrug_efflux_protein_bcr	multidrug	IL-13	Mediator	0.430	0.004
EOS	bicyclomycin-multidrug_efflux_protein_bcr	multidrug	IL-2	Mediator	0.463	0.002

EOS	bicyclomycin-multidrug_efflux_protein_bcr	multidrug	M20: Immune response_HMGB1 release from the cell	Transcriptome	-0.296	0.006
EOS	bicyclomycin-multidrug_efflux_protein_bcr	multidrug	M225: Eosinophil chemotaxis in asthma	Transcriptome	0.284	0.008
EOS	bicyclomycin-multidrug_efflux_protein_bcr	multidrug	M226: Proinflammatory cytokine production by eosinophils in asthma	Transcriptome	0.349	0.001
EOS	bicyclomycin-multidrug_efflux_protein_bcr	multidrug	M227: Mast cell migration in asthma	Transcriptome	0.279	0.010
EOS	bicyclomycin-multidrug_efflux_protein_bcr	multidrug	M23: GTP-XTP metabolism	Transcriptome	-0.283	0.009
EOS	DNA-binding_protein_H-NS	unclassified	IL-13	Mediator	0.446	0.003
EOS	DNA-binding_protein_H-NS	unclassified	IL-2	Mediator	0.475	0.001
EOS	DNA-binding_protein_H-NS	unclassified	M226: Proinflammatory cytokine production by eosinophils in asthma	Transcriptome	0.304	0.005
EOS	mdtL	multidrug	IL-5	Mediator	0.330	0.031
EOS	mdtL	multidrug	M222: Immune response_Differentiation of natural regulatory T cells	Transcriptome	0.301	0.005
EOS	mdtL	multidrug	M226: Proinflammatory cytokine production by eosinophils in asthma	Transcriptome	0.292	0.007
EOS	mdtL	multidrug	M23: GTP-XTP metabolism	Transcriptome	-0.350	0.001
EOS	mdtL	multidrug	M480: Role of inhibition of WNT signaling in the progression of lung cancer	Transcriptome	0.382	0.000
EOS	mdtM	multidrug	IL-13	Mediator	0.476	0.001
EOS	mdtM	multidrug	IL-2	Mediator	0.468	0.002
EOS	mdtM	multidrug	M226: Proinflammatory cytokine production by eosinophils in asthma	Transcriptome	0.328	0.002
EOS	omp36	multidrug	IL-13	Mediator	0.498	0.001

EOS	omp36	multidrug	IL-2	Mediator	0.519	0.000
EOS	omp36	multidrug	M226: Proinflammatory cytokine production by eosinophils in asthma	Transcriptome	0.293	0.006
EOS	ompF	multidrug	IL-13	Mediator	0.471	0.001
EOS	ompF	multidrug	IL-2	Mediator	0.511	0.000
EOS	ompF	multidrug	M20: Immune response_HMGB1 release from the cell	Transcriptome	-0.290	0.007
EOS	rifampin monooxygenase	rifamycin	IL-13	Mediator	0.429	0.004
EOS	rifampin monooxygenase	rifamycin	MIP-1d	Mediator	-0.455	0.002
EOS	rifampin monooxygenase	rifamycin	M10: Translation_Regulation of translation initiation	Transcriptome	-0.361	0.001
EOS	rifampin monooxygenase	rifamycin	M225: Eosinophil chemotaxis in asthma	Transcriptome	0.310	0.004
EOS	rifampin monooxygenase	rifamycin	M226: Proinflammatory cytokine production by eosinophils in asthma	Transcriptome	0.388	0.000
EOS	TEM-91	beta-lactam	IL-10	Mediator	0.408	0.007
EOS	TEM-91	beta-lactam	TARC	Mediator	-0.398	0.008
EOS	TEM-91	beta-lactam	M23: GTP-XTP metabolism	Transcriptome	0.326	0.002
EOS	TEM-91	beta-lactam	M27: CCR4-dependent immune cell chemotaxis in asthma and atopic dermatitis	Transcriptome	0.321	0.003
EOS	TolC	multidrug	IL-13	Mediator	0.440	0.003
EOS	TolC	multidrug	IL-2	Mediator	0.517	0.000
EOS	TolC	multidrug	M226: Proinflammatory cytokine production by eosinophils in asthma	Transcriptome	0.283	0.009
EOS	TolC	multidrug	M476: Glucocorticoid-induced elevation of intraocular pressure as glaucoma risk factor	Transcriptome	0.310	0.004
EOS	transcriptional regulatory protein CpxR	unclassified	MIP-1d	Mediator	-0.421	0.005

EOS	transcriptional regulatory protein CpxR	unclassified	M226: Proinflammatory cytokine production by eosinophils in asthma	Transcriptome	0.303	0.005
EOS	vanR	vancomycin	IL-13	Mediator	0.565	0.000
EOS	vanR	vancomycin	M225: Eosinophil chemotaxis in asthma	Transcriptome	0.304	0.005
EOS	vanR	vancomycin	M226: Proinflammatory cytokine production by eosinophils in asthma	Transcriptome	0.371	0.000
EOS	vanR	vancomycin	M227: Mast cell migration in asthma	Transcriptome	0.328	0.002
EOS	vanS	vancomycin	IL-13	Mediator	0.366	0.016
EOS	vanS	vancomycin	M225: Eosinophil chemotaxis in asthma	Transcriptome	0.286	0.008
EOS	vanS	vancomycin	M226: Proinflammatory cytokine production by eosinophils in asthma	Transcriptome	0.351	0.001

Table S6. List of bacterial species flagged as potential contaminants by being present in at least two of four reagent negative controls (relative abundance>0.001).

	**Guangzhou Blank1**	**Guangzhou Blank2**	**Shenzhen Blank1**	**Shenzhen Blank2**
Streptomyces lividans	0.004	0.000	0.002	0.000
Elizabethkingia anophelis	0.000	0.000	0.007	0.004
Elizabethkingia bruuniana	0.000	0.000	0.007	0.004
Elizabethkingia miricola	0.000	0.000	0.004	0.002
Elizabethkingia ursingii	0.000	0.000	0.004	0.002
Prochlorococcus marinus	0.020	0.018	0.000	0.000
Prochlorococcus sp. MIT 0604	0.001	0.000	0.003	0.000
Geobacillus genomosp. 3	0.000	0.000	0.002	0.001
Lactococcus lactis	0.000	0.000	0.004	0.003
Agrobacterium rhizogenes	0.012	0.006	0.000	0.000
Agrobacterium tumefaciens	0.008	0.005	0.000	0.000
Achromobacter denitrificans	0.001	0.000	0.001	0.000
Burkholderia stabilis	0.000	0.000	0.004	0.002
Paraburkholderia fungorum	0.000	0.000	0.031	0.015
Comamonas testosteroni	0.000	0.000	0.018	0.009
Variovorax sp. PMC12	0.000	0.000	0.002	0.001
Janthinobacterium sp. 1_2014MBL_MicDiv	0.000	0.000	0.003	0.001
Janthinobacterium sp. LM6	0.000	0.000	0.003	0.001
Aeromonas hydrophila	0.000	0.000	0.003	0.001
Cedecea neteri	0.000	0.000	0.023	0.011
Hafnia alvei	0.000	0.000	0.006	0.002
Serratia ficaria	0.000	0.000	0.003	0.001
Serratia fonticola	0.000	0.000	0.003	0.001
Serratia liquefaciens	0.000	0.000	0.022	0.010
Serratia marcescens	0.000	0.000	0.005	0.002
Serratia plymuthica	0.000	0.000	0.010	0.004
Serratia proteamaculans	0.000	0.000	0.024	0.010
Acinetobacter radioresistens	0.005	0.003	0.000	0.000
Pseudomonas azotoformans	0.000	0.000	0.004	0.002
Pseudomonas sp. JY-Q	0.000	0.000	0.032	0.016
Pseudomonas sp. NS1(2017)	0.001	0.000	0.002	0.000
Pseudomonas stutzeri	0.001	0.000	0.001	0.001
Pseudomonas tolaasii	0.021	0.004	0.006	0.003

References

1. Bafadhel M, McCormick M, Saha S, McKenna S, Shelley M, Hargadon B, Mistry V, Reid C, Parker D, Dodson P, Jenkins M, Lloyd A, Rugman P, Newbold P, Brightling CE. 2012. Profiling of sputum inflammatory mediators in asthma and chronic obstructive pulmonary disease. Respiration 83:36-44.
2. Murray PR, Washington JA. 1975. Microscopic and baceriologic analysis of expectorated sputum. Mayo Clin Proc 50:339-44.
3. Clarke EL, Taylor LJ, Zhao C, Connell A, Lee JJ, Fett B, Bushman FD, Bittinger K. 2019. Sunbeam: an extensible pipeline for analyzing metagenomic sequencing experiments. Microbiome 7:46.
4. Marcel M. 2011. Cutadapt removes adapter sequences from high-throughput sequencing reads. EMBnet Journal 17, 10-12.
5. Li H, Durbin R. 2009. Fast and accurate short read alignment with Burrows-Wheeler transform. Bioinformatics 25:1754-60.
6. Yin X, Jiang XT, Chai B, Li L, Yang Y, Cole JR, Tiedje JM, Zhang T. 2018. ARGs-OAP v2.0 with an expanded SARG database and Hidden Markov Models for enhancement characterization and quantification of antibiotic resistance genes in environmental metagenomes. Bioinformatics 34:2263-2270.
7. Wood DE, Lu J, Langmead B. 2019. Improved metagenomic analysis with Kraken 2. Genome Biol 20:257.
8. Wang Z, Yang Y, Yan Z, Liu H, Chen B, Liang Z, Wang F, Miller BE, Tal-Singer R, Yi X, Li J, Stampfli MR, Zhou H, Brightling CE, Brown JR, Wu M, Chen R, Shu W. 2020. Multi-omic meta-analysis identifies functional signatures of airway microbiome in chronic obstructive pulmonary disease. ISME J 14:2748-2765.
9. Kim D, Langmead B, Salzberg SL. 2015. HISAT: a fast spliced aligner with low memory requirements. Nat Methods 12:357-60.
10. Li B, Dewey CN. 2011. RSEM: accurate transcript quantification from RNA-Seq data with or without a reference genome. BMC Bioinformatics 12:323.
11. Love MI, Huber W, Anders S. 2014. Moderated estimation of fold change and dispersion for RNA-seq data with DESeq2. Genome Biol 15:550.
12. Langfelder P, Horvath S. 2008. WGCNA: an R package for weighted correlation network analysis. BMC Bioinformatics 9:559.
13. Wang F, Liang Z, Yang Y, Zhou L, Guan L, Wu W, Jiang M, Shi W, Deng K, Chen J, Chen R. 2019. Reproducibility of fluid-phase measurements in PBS-treated sputum supernatant of healthy and stable COPD subjects. Int J Chron Obstruct Pulmon Dis 14:835-852.
14. Lloyd-Price J, Arze C, Ananthakrishnan AN, Schirmer M, Avila-Pacheco J, Poon TW, Andrews E, Ajami NJ, Bonham KS, Brislawn CJ, Casero D, Courtney H, Gonzalez A, Graeber TG, Hall AB, Lake K, Landers CJ, Mallick H, Plichta DR, Prasad M, Rahnavard G, Sauk J, Shungin D, Vazquez-Baeza Y, White RA, 3rd, Investigators I, Braun J, Denson LA, Jansson JK, Knight R, Kugathasan S, McGovern DPB, Petrosino JF, Stappenbeck TS, Winter HS, Clish CB, Franzosa EA, Vlamakis H, Xavier RJ, Huttenhower C. 2019. Multi-omics of the gut microbial ecosystem in inflammatory bowel diseases. Nature 569:655-662.
15. Benjamini Y, Hochberg Y. 1995. Controlling the false discovery rate: a practical and powerful approach for multiple testing. J R Statist Soc B 57:289-300.
16. Peters MC, Ringel L, Dyjack N, Herrin R, Woodruff PG, Rios C, O'Connor B, Fahy JV, Seibold MA. 2019. A Transcriptomic Method to Determine Airway Immune Dysfunction in T2-High and T2-Low Asthma. Am J Respir Crit Care Med 199:465-477.
